# Identification of Different Classes of VOCs Based on Optical Emission Spectra Using a Dielectric Barrier Helium Plasma Coupled with a Mini Spectrometer

**DOI:** 10.1021/acsmeasuresciau.3c00066

**Published:** 2024-01-01

**Authors:** Jingqin Mao, Yahya Atwa, Zhenxun Wu, David McNeill, Hamza Shakeel

**Affiliations:** † School of Electronics, Electrical Engineering and Computer Science, Queen’s University Belfast, Belfast BT7 1NN, United Kingdom; ‡ Queen’s Business School, Queen’s University Belfast, Belfast BT7 1NN, United Kingdom

**Keywords:** volatile organic compounds, dielectric barrier helium plasma, optical emission spectra, photoionization detector, micro gas chromatography

## Abstract

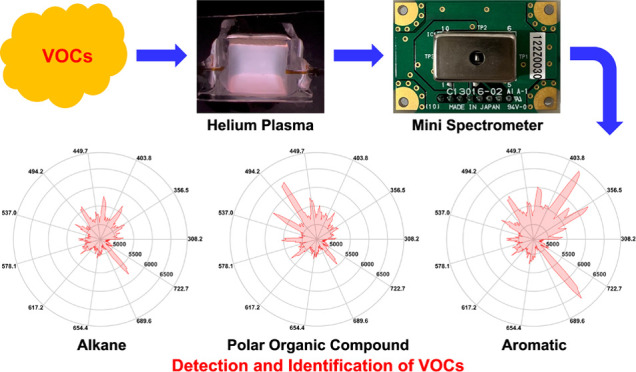

In this study, a micro helium dielectric barrier discharge (μHDBD) plasma device fabricated using 3D printing and molding techniques was coupled with a mini spectrometer to detect and identify different classes of volatile organic compounds (VOCs) using optical emission spectrometry (OES). We tested 11 VOCs belonging to three different classes (straight-chain alkanes, aromatics, and polar organic compounds). Our results clearly demonstrate that the optical emission spectra of different classes of VOCs show clear differences, and therefore, can be used for identification. Additionally, the emission spectra of VOCs with a similar structure (such as *n*-pentane, *n*-hexane, *n*-heptane, *n*-octane, and *n*-nonane) have similar optical emission spectrum shape. Acetone and ethanol also have similar emission wavelengths, but they show different line intensities for the same concentrations. We also found that the side-chain group of aromatics will also affect the emission spectra even though they have a similar structure (all have a benzene ring). Moreover, our μHDBD-OES system can also identify multiple compounds in VOC mixtures. Our work also demonstrates the possibility of identifying different classes of VOCs by the OES shape.

## 1 Introduction

Gas chromatography (GC) is one of the most reliable and commonly used techniques for detection of volatile organic compounds (VOCs).^1^ However, the traditional GC systems are usually bulky, energy-intensive, and expensive.^2^ In comparison, the micro gas chromatography (μGC) system is portable, energy-efficient, and low-cost. μGC system can also conduct on-site and real-time sample analysis.^3^ A typical μGC system consists of multiple components, including a carrier gas, preconcentrator for sampling, separation column, detector, micropump, microvalves, and computer software for data acquisition.^4,5^ Several μGC systems have been reported.^6−10^ Among these designs, μGC systems based on photoionization detectors (PIDs) demonstrated excellent performance for VOC detection.^11−13^ It is worth mentioning that the signal readout of most PIDs is based on an electrical signal readout. PIDs cannot identify VOCs without a separation column or mass spectrometer (MS).^14^ Although MS is usually bulky and expensive, it can identify VOCs based on the mass-to-charge ratio. Our group previously reported a colorimetric signal readout based on micro helium dielectric barrier discharge PID (μHDBD-PID). Our detector was based on detecting changes in image light intensity during injection of different VOCs.^15^ This work also required coupling of the detector with a GC column for the reliable identification of different compounds.

As a noninvasive and simple method, optical emission spectroscopy (OES) is often used in plasma diagnosis.^16^ OES can characterize interactions of species inside the plasma discharge both qualitatively and quantitatively.^17^ The operation of the OES needs an excitation source. Dielectric barrier discharge (DBD) plasma has the advantages of simple configuration, wide operating pressure range, low temperature and power consumption, zero electrode contamination, and high ionization ability.^18^ Therefore, DBD (argon- or helium-based) is often used as the plasma excitation source of OES, and several DBD-OES systems have been reported for detection of VOCs.^19−23^ Additionally, some studies have tried to use machine learning techniques to identify the type and concentration of VOCs.^16,24^ However, the reported DBD-OES systems usually have a relatively large size and need to be coupled with a bulky spectrometer, which makes them unsuitable for μGC systems.

In this work, we present a micro helium DBD-OES (μHDBD-OES) system for detection and identification of VOCs. To reduce the size of our μHDBD-OES system, we previously miniaturized the excitation source.^15^ The fabrication of the helium plasma chamber is based on using a combination of 3D printing, molding, and sintering processes. The final device dimensions are 5.4 mm × 5.2 mm × 5.0 mm (Length × Width × Height). We selected a commercially available mini spectrometer with dimensions of 20.1 mm × 12.5 mm × 10.1 mm (Length × Width × Height). Using the μHDBD-OES system, we performed emission spectra measurements on 11 VOCs from three different classes (straight-chain alkanes, aromatics, and polar organic compounds) and analyzed the differences between their emission spectra. All VOCs exhibited strong and clear emission spectra after direct injection into the plasma chamber. The emission spectra of the three different classes of VOCs are clearly distinguishable from each other. Moreover, we also conducted tests on different mixtures of VOCs. The results show that our μHDBD-OES system can clearly obtain and display the characteristic peaks of multiple VOCs in a single emission spectrum without using a GC column for separation, which makes the identification of a single VOC in a mixture possible. Our μHDBD-OES can provide a useful detection system for the future μGC system. Moreover, the size of the μGC system can be further reduced, as our system is capable of operating without the GC column. There still exist several implementation challenges that must be addressed in utilizing our μHDBD-OES system for detecting and identifying various classes of VOCs in conjunction with the μGC system. The primary challenge involves integrating the μHDBD-OES system and the μGC system. Another challenge is designing an integrated power supply capable of simultaneously powering the plasma chamber, mini spectrometer, and other μGC components, such as microvalves and micropumps.

## 2 Experimental Section

### 2.1 Materials

All 11 analytical-grade VOCs, including *n*-pentane, *n*-hexane, *n*-heptane, *n*-octane, and *n*-nonane (alkanes), ethanol (absolute), acetone and dichloromethane (polar compounds), and benzene, toluene, and ethylbenzene (aromatics), were purchased from Sigma-Aldrich (UK) and used as received. High-purity compressed helium gas cylinder (99.999% purity, grade zero, N5.0) was bought from BOC Gases (UK). Polydimethylsiloxane (PDMS) and hardener kit (SYLGARD 184) were purchased from Dow Silicones, UK. The Glassomer printable glass casting solution (UV-L50) and hardener used to fabricate the helium plasma chamber were bought from Glassomer GmbH (Germany). The transparent acrylic sheet used to make the ultraviolet (UV)-cured Glassomer plasma chamber was purchased from Rapid Electronics (UK). The fused silica capillary tubing (inner diameter of 150 μm) connecting the GC system and plasma chamber was purchased from Molex, UK. The silver conductive epoxy adhesive (Loctite Hysol 9492 and 8331 adhesive), which is used to connect electrodes to the plasma chamber electrode surfaces and heat sink compound was purchased from RS Components Ltd. (UK). The Norland Optical Adhesive 81 UV curable glue was ordered from Edmund Optics (UK). The circular ultrathin transparent glass slides (diameter of 30 mm and thickness of 0.17 mm) used for the observation window and plasma chamber electrode were purchased from Thermo Scientific (UK).

### 2.2 Experimental Setup

All VOC emission spectra measurements were conducted by a Hamamatsu C12880MA mini spectrometer coupled with a Hamamatsu C13016 evaluation board. Plasma videos and photos were recorded with an Apple iPhone 13 pro. VOCs were injected via an Agilent 7820A type GC system with an automatic pressure controller and split/splitless sample injection function. The image of a mini spectrometer slit was captured by a Keyence (VHX-7000) 4K high-accuracy digital microscope (UK).

**Figure 1:**
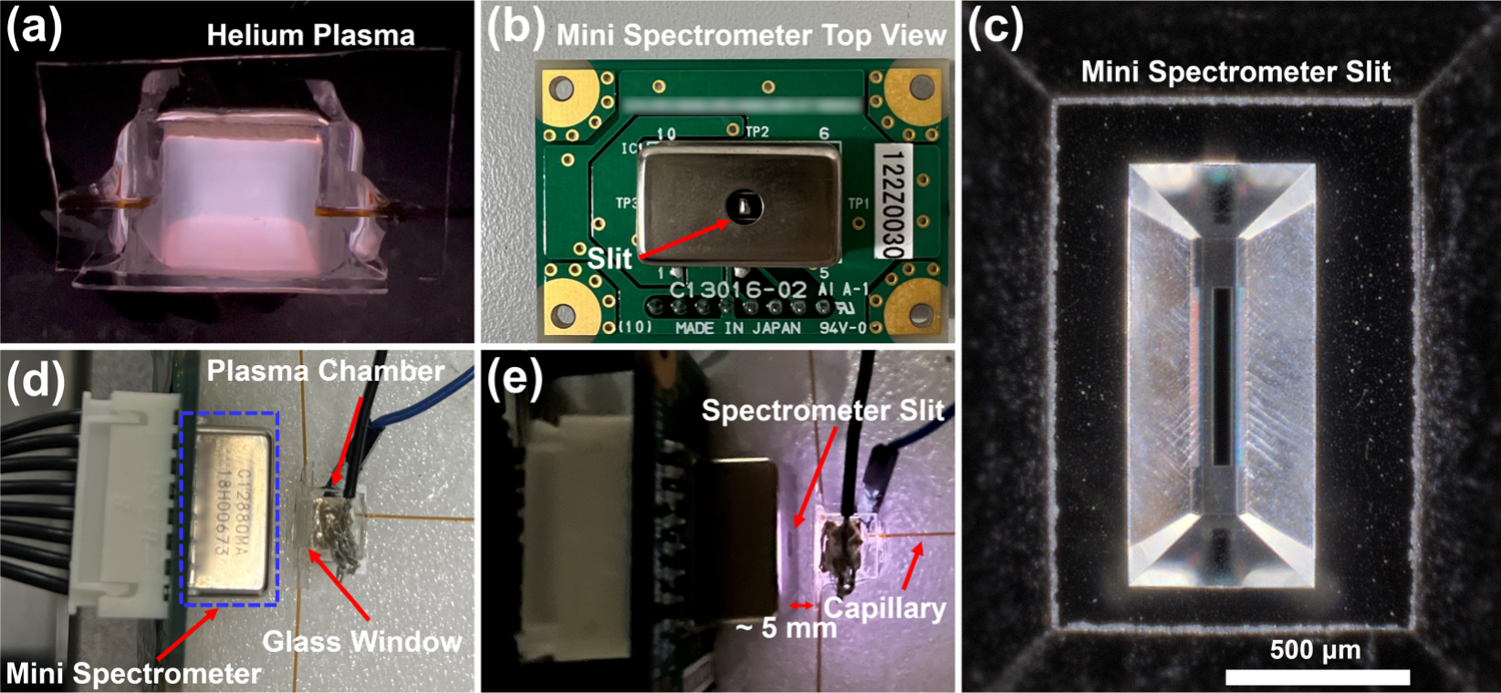
(a) Helium plasma under stable operation. (b) Hamamatsu C12880MA mini spectrometer, including the readout circuit board. (c) Optical image of mini spectrometer slit (magnification 50×). (d) Spectrometer and plasma chamber when the power supply is off. (e) Image of the mini spectrometer and plasma chamber when the power supply is turned on. The distance between the mini spectrometer slit and the glass observation window is approximately 5 mm.

The μHDBD-PID used in this work was fabricated and assembled following the same process reported in our earlier work and powered by the same power supply (Figures S1–S2).^15^ One key difference in our current study involves direct sample injection into the device using a fused silica capillary tube connected to an Agilent 7820A GC system. Figure 1a shows the helium plasma generated inside the chamber. The plasma is first operated for 10 min before each test to remove nonhelium gases (mainly nitrogen, oxygen, and water vapor) from the chamber. The mini spectrometer was mounted on the circuit board (Figure 1b), and the slit is located at the center of the spectrometer. Figure 1c shows a zoomed-in image of a mini spectrometer slit. The μHDBD was then placed directly in front of a mini spectrometer (Figure 1d) at a distance of approximately 5 mm. When helium plasma is generated, photons from the plasma chamber enter the mini spectrometer through the slit (Figure 1e). A shorter distance can improve photon efficiency, but it causes electrical breakdown of air and can damage the mini spectrometer since the outer shell of the spectrometer is metallic. The mini spectrometer is connected to the C13016 Hamamatsu evaluation board, and the collected spectra data is directly uploaded to the computer through a cable (Figure S2). All VOCs and VOC mixtures were injected directly into the plasma chamber through the GC injection port manually in the liquid form. We used high-purity helium as a carrier gas, and the GC system was set to split mode for all sample injections. The GC injection port inlet temperature and pressure were set to 270 °C and 20 Psi, respectively. The integration time of the mini spectrometer is set at 500 μs.

## 3 Results and Discussion

### 3.1 Helium Plasma Emission Spectra

**Figure 2:**
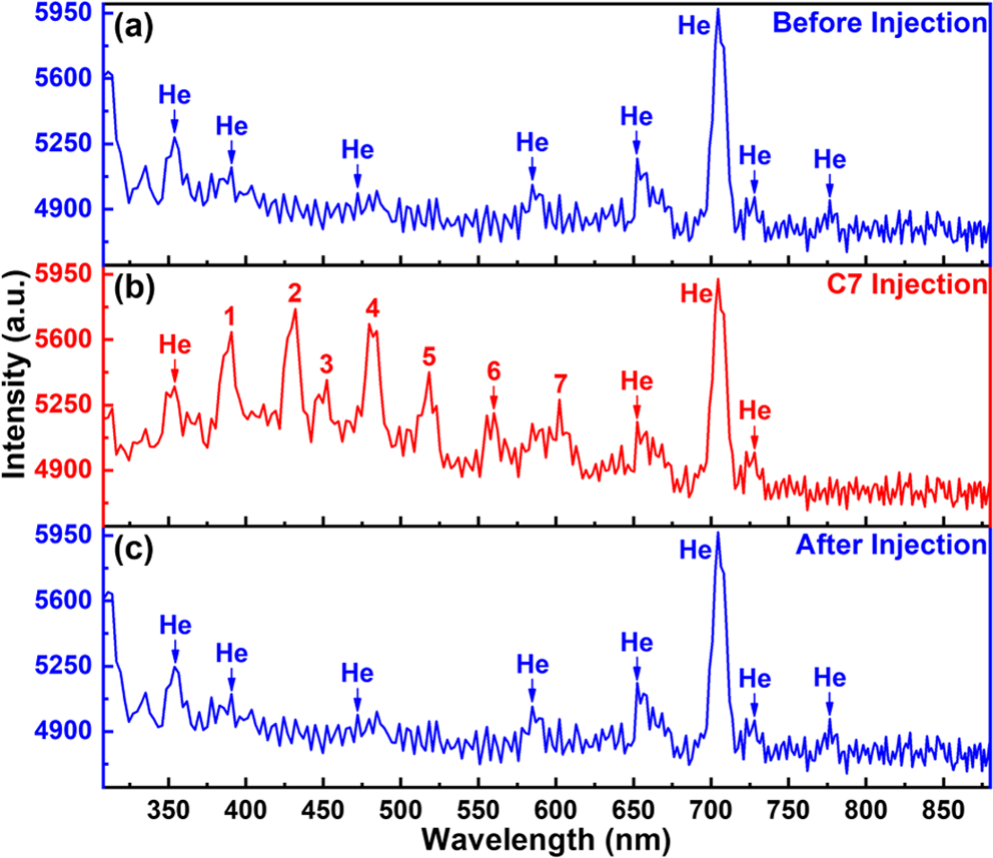
(a–c) Emission spectra change of helium plasma before and after C7 injection in the 308 to 880 nm range (sample injection volume of 0.2 μL and split ratio of 200:1). Peaks labeled 1 to 7 in panel (b) appear after injection of C7.

Figure 2 shows the helium plasma emission spectra before and after injection of *n*-heptane (C7) into the plasma chamber. We could see multiple emission peaks for helium plasma in the range of 308 to 880 nm. It is worth noting the reliable spectral response range of our spectrometers is between 340 and 850 nm.^25^ Although the spectrometer can still detect the signal outside the reliable range with reduced sensitivity, we mainly focus on the spectra lines distributed in the range of 340 to 850 nm. We also observed that the spectrometer signal usually has a large fluctuation when integration time is lower than 500 μs, which makes it difficult for us to obtain stable spectral results. Moreover, a longer integration time causes the merger of peaks with close emission peak wavelengths.

Before C7 injection, we measured a continuous helium plasma spectrum (Figure 2a), and the main peaks were located at 353.9 390.8, 472.1, 584.7, 652.4, 704.6, 728.1, and 776.6 nm (all data were rounded to one decimal place). Figure S3 shows a helium plasma spectrum with detailed peak values. The peak centered at 353.9 nm is broad, which can be attributed to the He emission line at 356 nm.^26,27^ Note that there is a minor difference between our spectrometer measurement results and the reported spectral line wavelengths. This difference is caused by the limited resolution of our mini spectrometer and merger of spectral peak lines. It is worth noting that there is an emission peak at 335.2 nm outside the reliable spectral response range, as well. This peak can also be attributed to the emission line of He at 336 nm.^26^ In the reliable spectral response range, the peaks concentrated at 390.8, 472.1, 584.7, and 728.1 nm are mainly associated with the He emission lines and also reported in the literature at 388.9, 471.3, 587.6, and 728.1 nm, respectively.^28−31^ As shown in Figure S3, there are four adjacent emission peaks from 652.4 to 668.4 nm. The peaks located at 652.4 and 654.4 nm are related to the He emission lines similar to the values reported in the literature in the range of 654 to 656 nm,^26,27^ and the peaks of 662.4 and 668.4 nm are contributed by the He emission lines at 667 or 667.8 nm.^26,29^ The emission line at 704.6 nm is the strongest before and after C7 injection, and this line corresponds to the He emission lines at 706.5 nm.^28,29,31^ Additionally, this peak also has a merged peak at 708.3 nm (Figure S3). For the peak centered at 776.6 nm, several studies related to He plasma emission spectrum identify it as the Oxygen (O) emission line (777.4 or 776 nm).^26,32^ However, our plasma does not come in direct contact with air, and the plasma chamber has been purged with a high-purity helium gas flow for 10 min before each test. More importantly, the intensity of this peak did not increase after the injection of VOCs containing oxygen atoms, such as acetone and ethanol (Figure 4). Besides, we also found this peak also appeared at similar positions in other related helium plasma studies.^22,27^ Therefore, we believe that this peak and its adjacent peak (781.3 nm; Figure S3) also belong to He emission.

The helium plasma emission spectrum clearly changed after C7 injection (Figure 2b). We could easily identify several emission peaks belonging to He plasma, but seven new emission peaks also appeared after C7 injection. These peaks are related to the species generated during the C7 ionization process in the He plasma. In addition, the ionization of C7 will also change the composition of He plasma and affect the distribution of the emission lines. After the injection of C7, the emission spectrum of He plasma returned to the original state (Figure 2c) in ∼5 s, which demonstrates that it is feasible to use the emission plasma spectrum as the sensing signal to detect VOCs. However, identification of different classes of VOCs required more tests. Therefore, we conducted comprehensive emission spectra measurements on all 11 VOCs.

### 3.2 Emission Spectra of Alkanes

**Figure 3:**
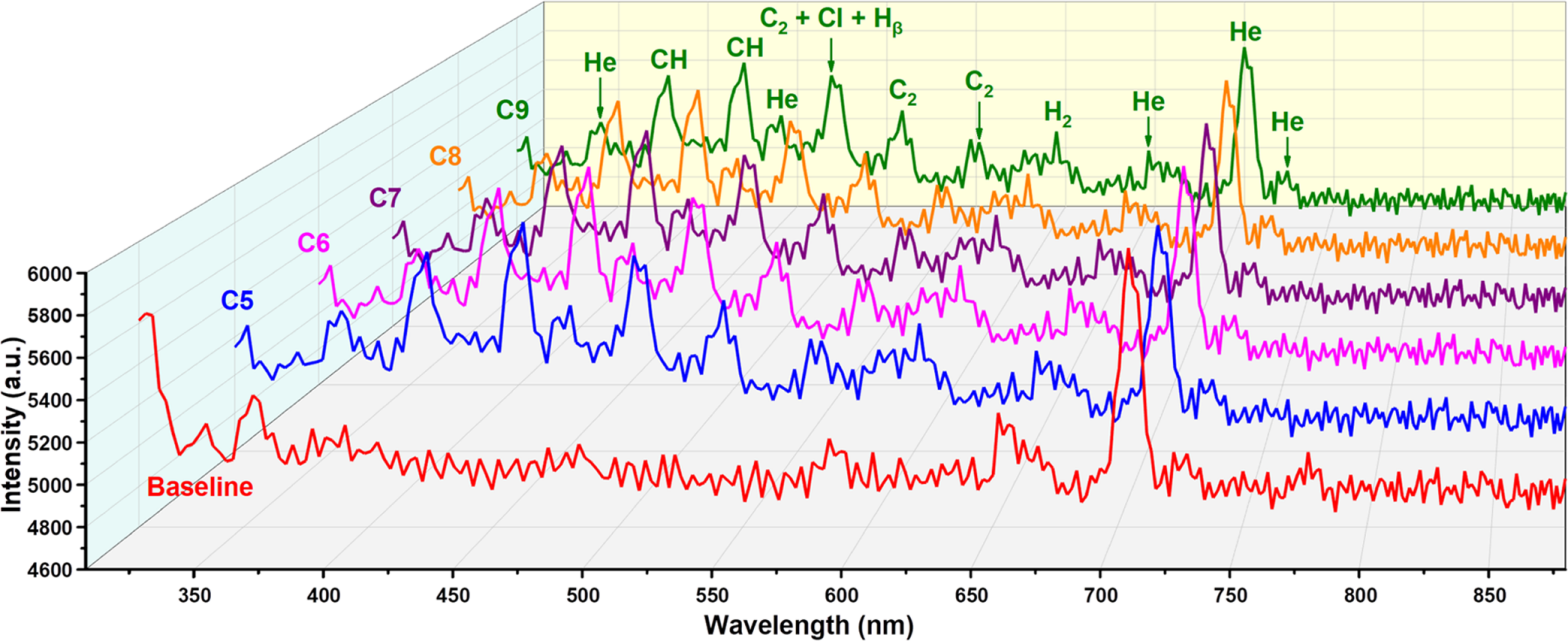
Emission spectra of He plasma during C5, C6, C7, C8, and C9 injection and He plasma baseline emission spectrum in the range of 308 to 880 nm. The injection volume is 0.2 μL with a split ratio of 200:1. The emission peaks were labeled according to species.

Figure 3 shows the plasma emission spectra after injection of *n*-pentane (C5), *n*-hexane (C6), *n*-heptane (C7), *n*-octane (C8), and *n*-nonane (C9). The emission spectra of C5–C9 have nearly the same peak shapes and peak distribution. A detailed distribution of peaks and values with comparison to the helium plasma spectrum is given in Figure S4 (C7 is used as an example). As shown in Figures 3 and S4, the two strong emission lines at 390.8 and 432.0 nm appeared in all alkane emission spectra and are caused by the excitation of CH (B^2^∑ → X^2^Π) and CH (A^2^Δ → X^2^Π) at 387 and 431 nm, respectively.^33,34^ The emission peak at 432.0 nm is the strongest line that appeared for all reported alkanes due to the abundance of the CH structure in alkane molecules. The peak at 452.2 nm and its adjacent peak at 447.2 nm are related to the He emission peak at 447.1 nm (4^3^D → 2^3^P).^28,31,35^ This peak is not obvious in the helium plasma emission spectrum, which indicates that the injection of alkanes also changed the species composition of the helium plasma and led to the change of the emission spectrum. The next peak is a broad peak because of three merged peaks (472.1, 479.5, and 484.4 nm). The peak centered at 472.1 nm, together with the following peaks at 518.2 and 560.0 nm, belong to the C_2_ Swan band (d^3^Π_g_ → a^3^Π_a_), which is caused by the triplet state C2 transitions between different vibrational bands (v′ = 1 – v" = 0:473 nm, v′ = 0 – v" = 0:516 nm, and v′ = 0 – v" = 1:563 nm).^33,34^ The peak located at 479.5 nm is contributed by CI (476.9 nm), and the peak at 484.4 nm corresponds to hydrogen Balmer lines (H_β_ at 486.1 nm).^32,36,37^ The H^+^ generated during the ionization of alkanes has the probability of combining and forming H_2_ molecules after collision, and H_2_ will be excited again. The peak at 602.2 nm is related to this process, and this peak corresponds to the Q1 and Q2 transitions (0–0, 601.83 nm, and 602.38 nm) of the H_2_ Fulcher-α system.^38^ During alkanes injection, there is no obvious change in the intensities of the He emission peak group starting from 652.4 nm (652.4, 656.4, 662.4, and 668.4 nm) and the peak at 728.1 nm. The intensity of the He emission peak at 704.6 nm is slightly decreased, and the peak group centered at 776.6 nm disappeared for all alkanes.

It is worth noticing that there is a significant decrease in emission line intensity (peak at 310.9 nm in Figure S4) in the near-ultraviolet (UV) range outside the reliable spectral range of our spectrometer. This change close to the UV region may correspond to the absorption of photons by alkanes and the resulting He plasma species change. The most important point to note is that the test results of five different alkanes show that our detection system can generate a stable emission spectrum for alkanes. Transitions corresponding to CH (B^2^∑ → X^2^Π), CH (A^2^Δ → X^2^Π) and C_2_ Swan band (d^3^Π_g_ → a^3^Π_a_) are the main contributing processes to the emission spectra of alkanes. These findings can make it possible to distinguish alkanes from other classes of compounds using the shape of the emission spectra. However, it is still difficult to separate alkanes from each other, since straight-chain alkanes have the same molecular structure (methyl and methylene).

### 3.3 Emission Spectra of Polar Organic Compounds

**Figure 4:**
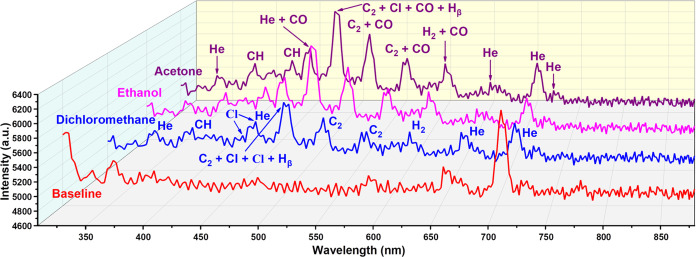
Emission spectra of He plasma during injection of acetone, ethanol, and dichloromethane injection (0.2 μL sample volume injected under a split ratio of 200:1) and He plasma baseline emission spectrum in the range of 308 to 880 nm. The emission peaks have been labeled according to species.

The emission spectra of acetone, ethanol, and dichloromethane in He plasma are presented together in Figure 4. The emission spectra peak distribution of polar organic compounds is different compared to alkanes. The peak centered at 479.5 nm is the strongest peak for all three polar organic compounds. Unlike alkanes, acetone contains CO in its molecular structure, and the corresponding wavelengths of CO emission line are centered around 450, 483, 519, 560, and 607 nm.^22,39,40^ Therefore, the intensity of the peaks around these positions will increase. As shown in Figures 4 and S5, the intensity of peaks centered at 452.2, 472.1 nm (479.5, 484.4 nm), 518.2, 560.0, and 602.2 nm is higher compared to alkanes (Figures 3 and S4). In particular, the contribution of CO in the molecular structure makes the merged peak centered at 479.5 nm the highest intensity peak based on C_2_, CI, and H_β_. The intensity of the two peaks associated with CH (B^2^∑ → X^2^Π) and CH (A^2^Δ → X^2^Π) at 387 and 431 nm is also reduced. The emission spectrum distribution of ethanol is like acetone due to the CO (single bond) in its molecular structure. The intensity of peaks centered at 452.2, 472.1 nm (479.5 and 484.4 nm), 518.2, 560.0, and 602.2 nm for ethanol is also higher compared to alkanes. However, the intensity of these peaks is lower than that for acetone (Figures 4 and S5–S6). The intensity of the two peaks associated with CH (B^2^∑ → X^2^Π) and CH (A^2^Δ → X^2^Π) at 387 and 431 nm for ethanol has the same level as acetone (Figures S5–S6). It should be noted that our mini spectrometer did not capture the strong emission peak around 310 nm associated with the OH group for ethanol, as reported in published work.^32−34^ In contrast, the peak intensity in the near-UV region is low, and it is the same for all three polar organic compounds (Figure 4). As mentioned above, VOCs will absorb photo energy during ionization, and the photons corresponding to the UV region have higher energy. The photons in the near-UV region emitted by OH are likely to have been absorbed by VOCs before they are recorded by the mini spectrometer. Additionally, there is also a strong possibility that the spectrometer did not respond to emission peaks near the UV region since it is outside the stated spectral range.

Dichloromethane contains chlorine atoms in its molecular structure, which makes its emission spectrum clearly different from those of both acetone and ethanol. As shown in Figures 4 and S5–S7, the intensity of the dichloromethane emission peak is obviously lower than that of acetone and ethanol. Dichloromethane also contains a CH structure (CH_2_Cl_2_), so the merged peak centered at 479.5 nm associated with C_2_, CI, and H_β_ is still the highest peak. This peak has a clear split compared to the peaks of acetone and ethanol. This is because the emission line related to Cl II is centered at 481.9 nm, and this peak also merged with C_2_, CI, and H_β_.^41,42^ Additionally, two Cl atomic lines at 439.0 and 452.6 nm were also detected by our mini spectrometer and recorded as 439.6 and 452.2 nm, respectively (Figure S7).^41,42^ For CH (B^2^∑ → X^2^Π) and CH (A^2^Δ → X^2^Π) at 387 and 431 nm, we only recorded one weak CH peak at 390.8 nm, which demonstrates another difference between dichloromethane and other two polar organic compounds. Dichloromethane has a complete C_2_ Swan band (d^3^Π_g_ → a^3^Π_a_) and H_2_ emission peak, but the intensity of these peaks is much lower than that of acetone and ethanol (Figure S7). Due to the poor sensitivity of the spectrometer in the near-infrared region, we did not observe the Cl emission peak at 833, 838, or 858 nm.^33^

The He plasma emission spectrum also changed during the injection of polar organic compounds. In addition to the new He emission peaks appearing at 447.2 and 452.2 nm, most of the He emission peaks (353.9, 652.4, and 728.1 nm) remained at the same intensity level as those without injection, as shown in Figure 4. The intensity of the helium line emission peak at 704.6 nm is significantly decreased, and the He peak group centered at 776.6 nm also disappeared for all three polar organic compounds. Although there are many differences in the emission spectra of acetone, ethanol, and dichloromethane, it is still easy to see that their emission spectra have roughly the same pattern. Moreover, their pattern is clearly different from the emission spectra of alkanes, which indicates the possibility of identifying different classes of VOCs from each other simply by the shape of the spectra rather than complicated spectral analysis.

### 3.4 Emission Spectra of Aromatic Compounds

**Figure 5:**
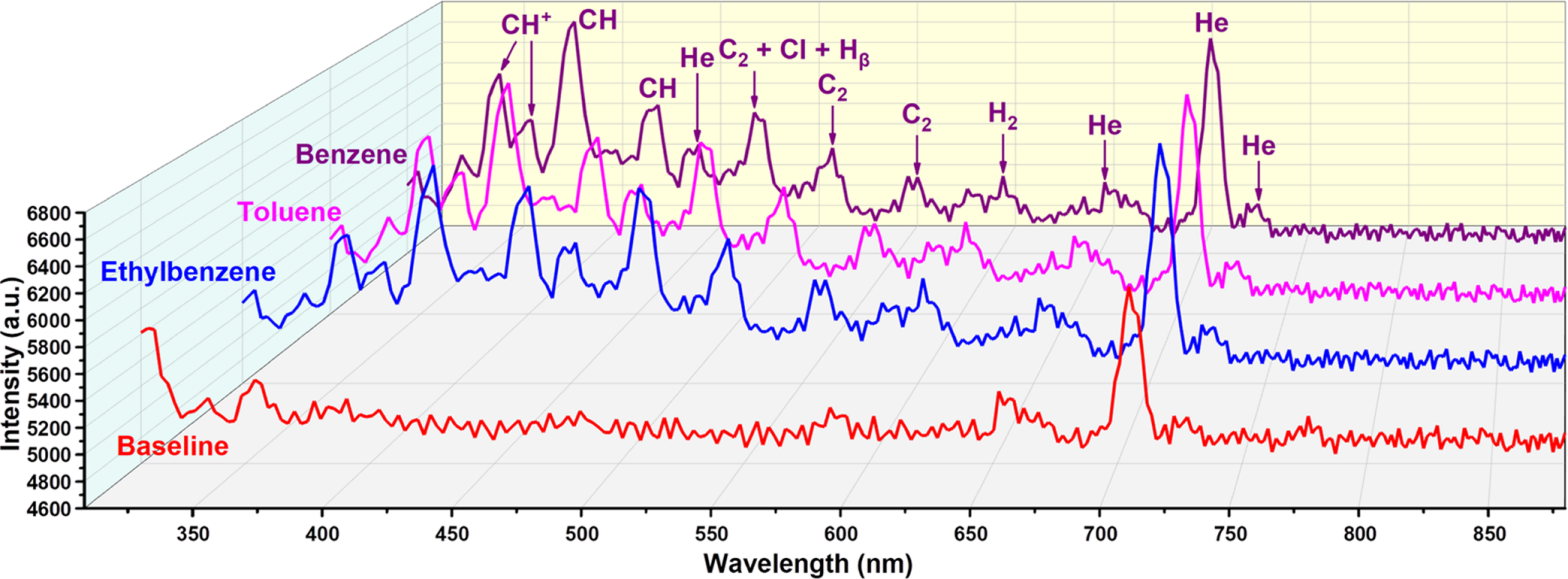
Emission spectra of He plasma during injection of benzene, toluene, and ethylbenzene (0.2 μL sample injection under a split ratio of 200:1) and He plasma baseline emission spectrum in the range of 308 nm to 880 nm. The emission peaks have been labeled according to species.

As shown in Figure 5, the emission spectra of aromatic compounds are different compared to those of alkanes and polar organic compounds. For aromatics, two new high-intensity peaks were observed at 353.9 and 369.8 nm, which are mainly dominated by the emission peaks of CH^+^ in the 350 to 370 nm range.^43,44^ Furthermore, the intensity of these two peaks gradually decreases from benzene to toluene to ethylbenzene, which indicates the presence of side-chain groups that negatively affect the emission of CH^+^. The peaks associated with CH (B^2^∑ → X^2^Π) and CH (A^2^Δ → X^2^Π) at 390.8 and 432.0 nm were also recorded, and the peak at 390.8 nm is the highest peak in all three aromatic compounds. Moreover, the peak intensity at 390.8 nm also decreases, while the peak intensity at 432.0 nm increases with the increase of the side-chain group (from benzene to benzene-CH_2_ and benzene-CH_2_CH_3_). The C_2_ Swan band (d^3^Π_g_ → a^3^Π_a_) was also observed in all three aromatics. It is worth mentioning that the intensity of the three peaks (peak groups) where C_2_ Swan bonds are located (479.5, 518.2, and 560.0 nm) and H_2_ peak at 602.2 nm gradually decreases from ethylbenzene to toluene and benzene. The emission spectrum of benzene (Figure S8) is obviously different compared with alkanes. However, after the introduction of the side-chain group, the emission spectra of toluene and ethylbenzene became closer to the shape of alkanes (Figures 3 and 5). The emission spectra shape differences not only demonstrate the effect of side-chain groups on the emission spectra of aromatics but also reveal a potential method for identifying different aromatics. There is no big change in intensity for He emission peaks (peak group) at 452.2, 652.4, and 728.1 nm. But the intensity of the He emission peak at 704.6 nm is significantly increased, which is different from alkanes and polar organic compounds. The peak group centered at 776.6 nm also disappeared in the emission spectra of all three aromatic compounds. Finally, despite the minor differences in emission spectra of the three aromatic compounds, the overall pattern is similar, and it is obviously different from the overall shapes of alkanes and polar organic compounds (Figures 3–5).

### 3.5 Identification of Different Classes of VOCs

Table 1 lists the emission peak wavelengths and the corresponding species of He plasma and VOCs. C7 was selected as the representative of alkanes due to the high structural similarity of C5–C9. Benzene, toluene, and ethylbenzene also have a similar molecular structure (all contain a benzene ring) and the same emission spectrum peak distribution, so benzene was selected as the representative of aromatic compounds. Acetone, ethanol, and dichloromethane are listed separately due to notable differences (C=O, OH, and Cl) in structure, even though all three are polar compounds. As shown in Table 1, each peak wavelength (group) corresponds to one or more species.

**Table 1:** Recorded Wavelength of He and the OES Peaks of Target VOCs^a^

**analytes**	**recorded OES peak wavelength (nm)**	**corresponding species**	**refs**
He	353.9, 390.8, 472.1, 584.7, 652.4 704.6, 728.1, 776.6	He	(22,26−32)
C7 (representative straight chain alkane)	390.8, 432.0	CH	(33,34)
	479.5	CI	(36)
	472.1, 518.2, 560.0	C_2_	(33,34)
	484.4	H_β_	(32,36,37)
	602.2	H_2_	(38)
acetone	390.8, 432.0	CH	(33,34)
	479.5	CI	(36)
	472.1, 518.2, 560.0	C_2_	(33,34)
	452.2, 484.4, 518.8, 560.0, 602.2	CO	(22,39,40)
	484.4	H_β_	(32,36,37)
	602.2	H_2_	(38)
ethanol	390.8, 432.0	CH	(33,34)
	479.5	CI	(36)
	472.1, 518.2, 560.0	C_2_	(33,34)
	452.2, 484.4, 518.8, 560.0, 602.2	CO	(22,39,40)
	484.4	H_β_	(32,36,37)
	602.2	H_2_	(38)
dichloromethane	390.8, 432.0	CH	(33,34)
	479.5	CI	(36)
	472.1, 518.2, 560.0	C_2_	(33,34)
	484.4	H_β_	(32,36,37)
	602.2	H_2_	(38)
	439.6	Cl I	(41,42)
	452.2	Cl I	(41,42)
	482.0	Cl II	(41,42)
benzene (representative aromatic compound)	353.9, 369.8	CH^+^	(43,44)
	390.8, 432.0	CH	(33,34)
	479.5	CI	(36)
	472.1, 518.2, 560.0	C_2_	(33,34)
	484.4	H_β_	(32,36,37)
	602.2	H_2_	(38)

a The corresponding species were measured by a μHDBD-OES system. Note that one wavelength may be associated with multiple species due to their close emission wavelengths and merger of peaks.

Using either peak intensity difference or specific component emission peak is an effective method to identify VOCs, as reported.^33,34^ Moreover, MS is required to determine the molecular structure of the target sample more accurately. However, rapid detection and identification of VOCs are essential for the μGC system. Currently, our device can easily detect the presence of VOCs. Therefore, we propose a method for identifying different classes of VOCs. Our idea is to identify different classes of VOCs using the shape of their emission spectra.

**Figure 6:**
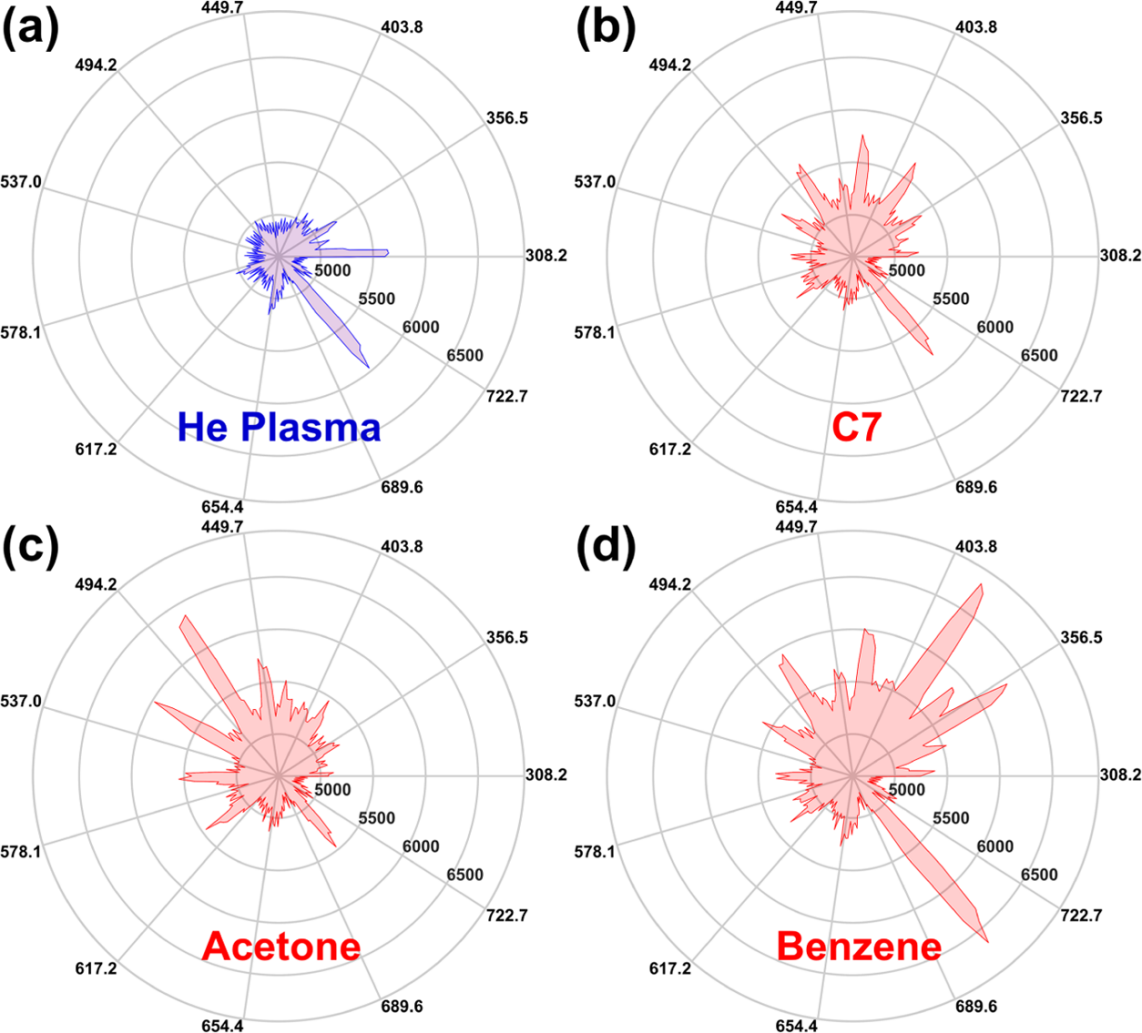
Radar plots of emission spectra from 308.2 to 752.2 nm corresponding to Figures 3–5. (a) He plasma, (b) C7, (c) acetone, and (d) benzene. The ring axis corresponds to wavelength (nm) and the polar axis to intensity (a.u.).

Figures 3–5 present the emission spectra of 11 VOCs from three different classes (alkanes, polar, and aromatics). There are no obvious peaks after 752.2 nm for all 11 VOCs. Therefore, we plot the emission spectra in the range of 308.2 to 752.2 nm (wavelength values rounded to one decimal place) as radar charts to clearly demonstrate the difference in the shape of emission spectra. Figure 6 shows the emission spectra as radar plots of He plasma, C7, acetone, and benzene ranging from 308.2 to 752.2 nm. It is easier to see the emission spectrum shape differences between different types of VOCs and He plasma through these radar charts. Additionally, the polar axis (intensity) of all radar charts is set to the same scale. Therefore, the emission spectra size reflects the overall peak intensity. The emission intensity of He plasma is at a relatively low level before the injection of VOCs (Figure 6a). A typical emission spectrum shape of alkanes as a radar plot is shown in Figure 6b. CH-related peaks, C_2_ Swan band (d^3^Π_g_ → a^3^Π_a_), and the He peak at 704.6 nm mainly contribute to the final shape. Additionally, we have shown previously in Figure 3 that the emission spectrum shape and size of all target alkanes (C5, C6, C8, and C9) are very similar to C7. The emission spectrum shape of acetone (Figure 6c) is obviously different from that of alkanes due to the presence of CO in its molecular structure. Since the CO-related emission peak positions are close to C_2_, H_β_, and H_2_, these peaks are strengthened, and this increases the overall intensity of the acetone emission spectrum. The molecular structure of ethanol is different from acetone, but as both compounds contain oxygen-containing groups (CO, OH), ethanol has a similar spectrum shape (Figure S9a) compared to acetone (Figure 6c). The emission spectrum shape of dichloromethane (Figure S9b) is different from those of acetone and ethanol due to the differences in atomic composition. The emission spectra intensity of aromatic compounds is the largest among the three classes of VOCs used in this study, especially benzene (Figure 6d). The two strong peaks at 353.9 and 390.8 nm are the signature peaks of aromatic compounds, making their spectra shapes easily distinguishable. Toluene and ethylbenzene have similar emission spectra shapes compared to benzene (Figure 6d), as shown in Figure S9c–d. Moreover, the spectra of toluene and ethylbenzene gradually become like alkanes due to the introduction of alkane-based side chains, as discussed above.

The radar plots provided in Figures 6 and S9 clearly demonstrate the possibility of directly identifying different classes of VOCs by using the shapes of the emission spectra. It is important to note that an extensive emission spectra database is required to identify different classes of VOCs quickly. However, it is still difficult to identify a single VOC by looking at the shape of emission spectra, such as identifying straight-chain alkanes from each other and distinguishing acetone from ethanol. There is also a strong case to distinguish different aromatic compounds based on the subtle differences in emission spectra peak intensity caused by side-chain groups. But detailed identification of individual VOCs still requires the use of other analytical techniques, such as MS and IR spectroscopy.

### 3.6 Limit of Detection (LoD) and Linearity Range

**Figure 7:**
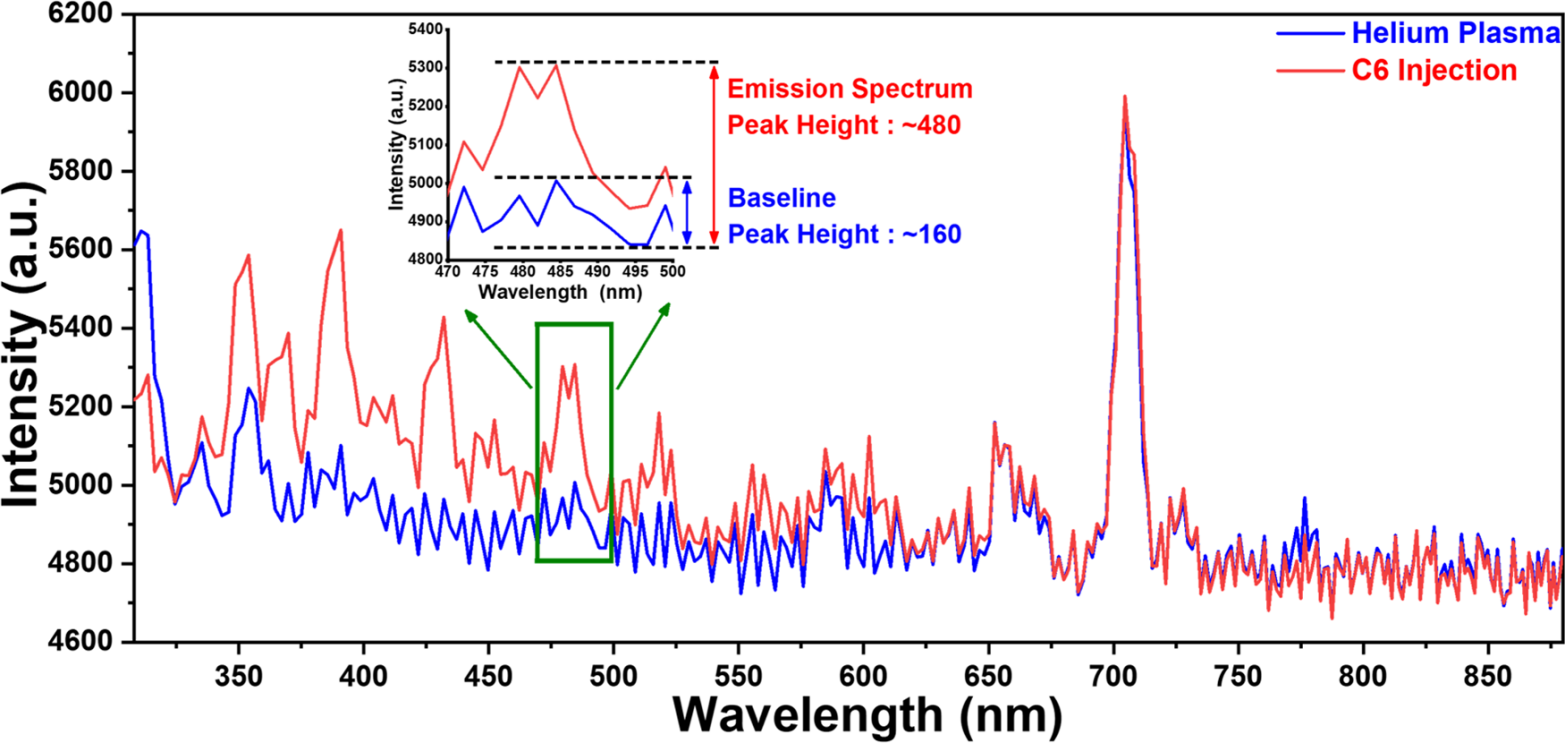
Limit of detection is defined as three times the baseline emission peak height. For example, the limit of detection for C6 is 74 ng.

The emission lines of He plasma are continuous, with a small fluctuation observed in the baseline. However, multiple emission peaks appeared during the injection of VOCs. After performing several tests, the peak group centered at 479.5 nm (472.1 and 484. 4 nm) was selected for the measurement of limit of detection (LoD) due to its fast response and high sensitivity for all VOCs. Besides, it can provide a wider linear range for most of the VOCs. The LoD was calculated as three times the He plasma baseline fluctuation of the peak group centered at 479.5 nm.^33^Figure 7 shows the emission spectrum change when 74 ng of C6 was injected into the plasma chamber compared with the He plasma baseline. The absolute peak intensity height (taken at the bottom of the He plasma baseline peak group at 489.5 nm for calculation) of the peak group at 479.5 nm is ∼480. The maximum peak intensity fluctuation of the He plasma baseline peak group here is ∼160; therefore, the calculated signal-to-noise ratio is ∼3.

Three devices were fabricated, and the LoD tests were conducted for all 11 VOCs to evaluate device-to-device variations. Table 2 presents the calculated average values of LoD with standard deviation (*n* = 3) between three different devices. Among the three classes of VOCs, the LoDs of alkanes are the highest, while the aromatic compounds have the lowest LoDs. For polar organic compounds, acetone and ethanol also have LoDs relatively lower than alkanes. However, dichloromethane has the highest LoD among the 11 tested compounds, which can be attributed to its low emission spectra peak intensity during injection (Figure 4). In contrast, benzene has the lowest detection limit among all tested VOCs due to its highest emission spectral intensity during injection (Figure 5).

**Table 2:** Summary of Limit of Detections with Standard Deviation (*n* = 3), Boiling Points, Ionization Potentials, and Linearity Ranges of 11 VOCs^8,45,46^^,^^a^

**compound**	**LoD (ng)**	**standard deviation** (*n* = 3)	**boiling point (°C)**	**IP (eV)**	**linearity range (ng)**
C5	83	7	36.06	10.35	83–626
C6	74	7	68.72	10.18	74–655
C7	80	4	98.38	10.08	80–855
C8	82	8	125.62	9.82	82–879
C9	81	7	150.8	9.71	81–1077
benzene	19	2	80.08	9.25	19–175
toluene	27	4	110.6	8.82	27–173
ethylbenzene	24	6	136.2	8.77	24–217
ethanol	46	9	78.24	10.43	46–592
acetone	33	5	56.08	9.69	33–588
dichloromethane	144	16	39.8	11.35	144–998

a The LoD values shown in the table are the average values based on the measurement of the three devices.

The linearity tests for 11 VOCs were also conducted and listed in Table 2. Due to the small volume of our plasma chamber and limited He plasma ionization capacity, a higher injection volume saturates our device. Therefore, we see no increase in emission spectra intensity after injection of a certain volume. We also observed a decreasing peak intensity when an excessive sample is injected. We have also observed that VOCs with a high emission spectrum intensity easily saturate the device and result in narrow linearity ranges. As shown in Table 2, VOCs with relatively high emission spectrum intensity usually have narrow linearity ranges, such as aromatics, acetone, and ethanol. VOCs that are difficult to ionize and therefore generate high-intensity emission peaks generally have wider linearity ranges, such as alkanes and dichloromethane.

The distance between the mini spectrometer slit and the plasma chamber observation window is the main factor limiting the improvement of LoDs. The current safe distance to prevent electrical breakdown from damaging the spectrometer is ∼5 mm. It is difficult to further reduce the distance since the mini spectrometer has a metal-based cover. Using an optical fiber-coupled mini spectrometer could help to achieve a better LoD.

### 3.7 Performance Evaluation of the Detection System with Multiple Compounds

**Figure 8:**
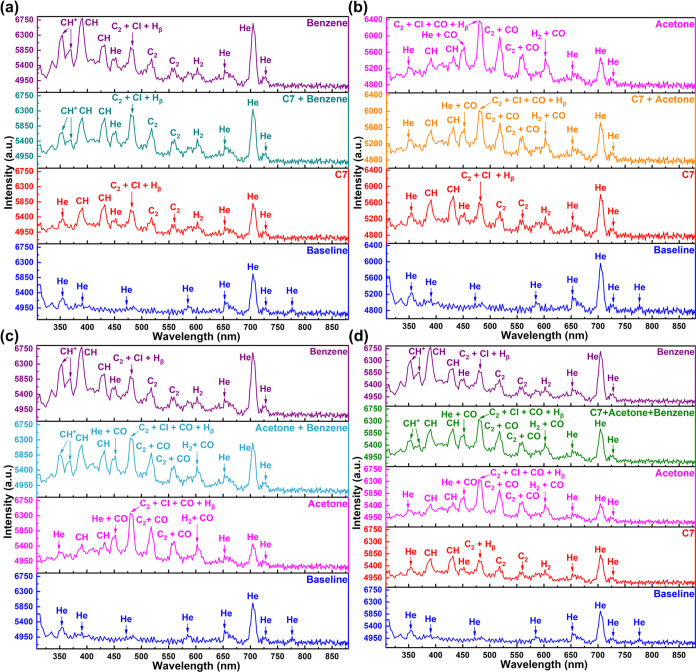
Emission spectra of VOCs mixtures. (a) Emission spectra of C7, benzene, and a mixture of C7 and benzene (volume ratio 1:1, injection volume is 0.2 μL under a split ratio of 200:1) and He plasma baseline emission spectrum. (b) Emission spectra of C7, acetone, and a mixture of C7 and acetone (volume ratio 1:1, injection volume is 0.2 μL under a split ratio of 200:1) and He plasma baseline emission spectrum. (c) Emission spectra of acetone, benzene, and a mixture of acetone and benzene (volume ratio 1:1, injection volume is 0.2 μL under a split ratio of 200:1) and He plasma baseline emission spectrum. (d) Emission spectra of C7, acetone, benzene, and a mixture of C7, acetone, and benzene (volume ratio 1:1:1, injection volume is 0.2 μL under a split ratio of 200:1). The emission spectra of C7, acetone, and benzene here are sourced from Figures 3 to 5. The emission spectra intensity decreases from the top to bottom in each graph. The emission peaks have been labeled according to species.

The detection performance of VOC mixtures for our proposed system was also evaluated by injecting four different mixtures. C7, acetone, and benzene were chosen to create different mixtures of VOCs. Figure 8a shows the emission spectrum of the C7 and benzene mixture (volume ratio 1:1) and is compared with the C7, benzene, and He plasma emission spectra. The overall intensity of the emission spectrum of the mixture is between C7 and benzene. Common peaks of C7 and benzene such as CH-related peaks (B^2^∑ → X^2^Π and A^2^Δ → X^2^Π), the C_2_ Swan band (d^3^Π_g_ → a^3^Π_a_), and the H_2_ emission peak were observed in the emission spectrum of the mixture. But the peak intensity of the mixture is different from individual compounds (C7 and benzene). The benzene CH peak intensity is much higher than C7, which leads to the CH peak intensity of the mixture becoming higher than C7 but lower than benzene. The intensity of the C_2_ Swan band and H_2_ emission peaks slightly increased compared to individual compounds (C7 and benzene). Additionally, two new peaks related to CH^+^ appeared at 353.9 and 369.8 nm, respectively. These peaks are the signature peaks that can be used to identify benzene. Another notable change is related to the He emission peak at 704.6 nm. As shown in Figure 8a, the peak at 704.6 nm was lower and higher than the He baseline for C7 and benzene injections, respectively. The intensity of this line is between C7 and benzene for the mixture.

It is easy to identify the presence of acetone by the strong peak group centered at 479.5 nm in the emission spectrum of a C7/acetone mixture (Figure 8b). CH-related peaks (B^2^∑ → X^2^Π and A^2^Δ → X^2^Π), C_2_ Swan band (d^3^Π_g_ → a^3^Π_a_), and H_2_ emission peak were also observed in the emission spectrum of the mixture. The C_2_ Swan band and H_2_ emission peak were reinforced due to the presence of CO (450, 483, 519, 560, and 607 nm). The intensity of CH emission peaks and baseline He peak at 704.6 nm remains between C7 and acetone.

Benzene and acetone are VOCs with a relatively high emission spectra intensity with obvious characteristic peak groups. In the emission spectrum of their mixture, the two strong peaks related to CH^+^ (353.9 and 369.8 nm) and another two strong peaks (390.8 and 432.0 nm) corresponding to CH are mainly contributed by benzene (Figure 8c). The highest peak group centered at 479.5 nm is mainly caused by acetone. C_2_ Swan band (d^3^Π_g_ → a^3^Π_a_) and H_2_ emission peak were also strengthened by acetone. Individual injections of benzene and acetone significantly increase and decrease the He emission peak intensity at 704.6 nm, respectively. Therefore, the He peak at 704.6 nm intensity in the mixture is between them.

Apart from the mixture of two VOCs, we also tested the emission spectrum of a C7, acetone, and benzene mixture (Figure 8d). The characteristic peaks of benzene and acetone can also be easily identified in the emission spectrum of the mixture. There are no characteristic peaks of C7, so identification of C7 requires a comparison with Figure 8c. The addition of C7 reduced the intensity of the CH^+^ and CH emission peaks associated with benzene. In addition, C7 weakens the intensity of CO from acetone on the C_2_ Swan band (d^3^Π_g_ → a^3^Π_a_) and H_2_ emission peak. C7 also reduces the He emission peak intensity at 704.6 nm.

In conclusion, the spectra of different sets of mixtures demonstrated that our system could identify multiple compounds in a mixture. It is necessary to build a comprehensive spectral database of single VOCs before the individual compounds can be identified in a mixture. It is clear that identification of various classes of VOCs can be realized through characteristic peaks and emission spectra comparison.

## 4 Conclusions

In this paper, an μHDBD-OES system utilizing emission spectra is used to detect and identify different classes of VOCs. The use of a mini spectrometer makes our system portable, which is suitable for an μGC system. The emission spectra measurements of alkanes, aromatics, and polar organic compounds show that each of these types has a different emission spectrum shape, which demonstrates the possibility of identifying VOC classes with the same atomic components. In addition, our μHDBD-OES system is also capable of detecting and identifying VOCs in a mixture. Specific VOCs can be easily identified based on characteristic peaks.

## Supporting Information Available

Final assembled helium plasma chamber (Figure S1); photo of μHDBD-OES system (Figure S2); optical emission spectrum of helium plasma (Figure S3); optical emission spectrum of C7, acetone, ethanol, dichloromethane, and benzene injected into plasma chamber (Figures S4–S8); and emission spectra radar plots of ethanol, dichloromethane, toluene, and ethylbenzene (Figure S9) (PDF)
