# Supplementary material for: Identification of Different Classes of VOCs Based on Optical Emission Spectra Using a Dielectric Barrier Helium Plasma Coupled with a Mini Spectrometer
Source: ACS Meas Sci Au. 2024 Jan 1;4(2):201–12. doi: 10.1021/acsmeasuresciau.3c00066 (PMC11027204; doi:10.1021/acsmeasuresciau.3c00066)
Supplement: Supplementary file 1 — tg3c00066_si_001.pdf [file tg3c00066_si_001.pdf]

Supporting Information for
Identification of different Classes of VOCs based on Optical Emission Spectra using a Dielectric Barrier Helium Plasma Coupled with a Mini Spectrometer

Jingqin Mao[†], Yahya Atwa[†], Zhenxun Wu[§], David McNeill[†], and Hamza Shakeel[†,*]

† School of Electronics, Electrical Engineering and Computer Science, Queen's University Belfast, Belfast, BT7 1NN, United Kingdom

§ Queen's Business School, Queen's University Belfast, Belfast, BT7 1NN, United Kingdom

* Corresponding Author, Email address: H.Shakeel@qub.ac.uk

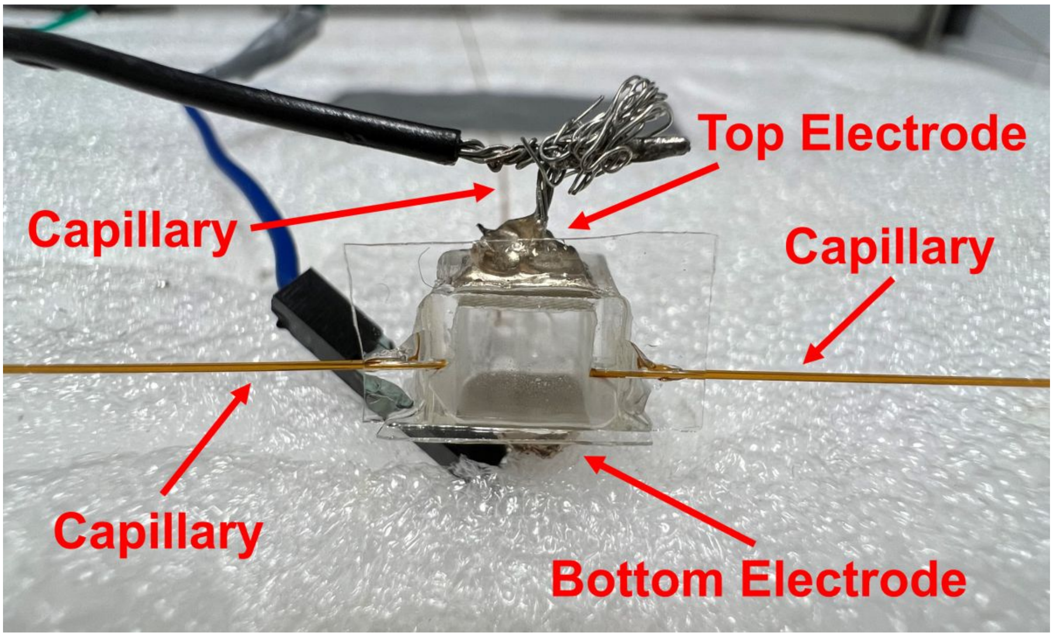

Figure S1. Final assembled helium plasma chamber using capillary tubes as gas inlet and outlets.

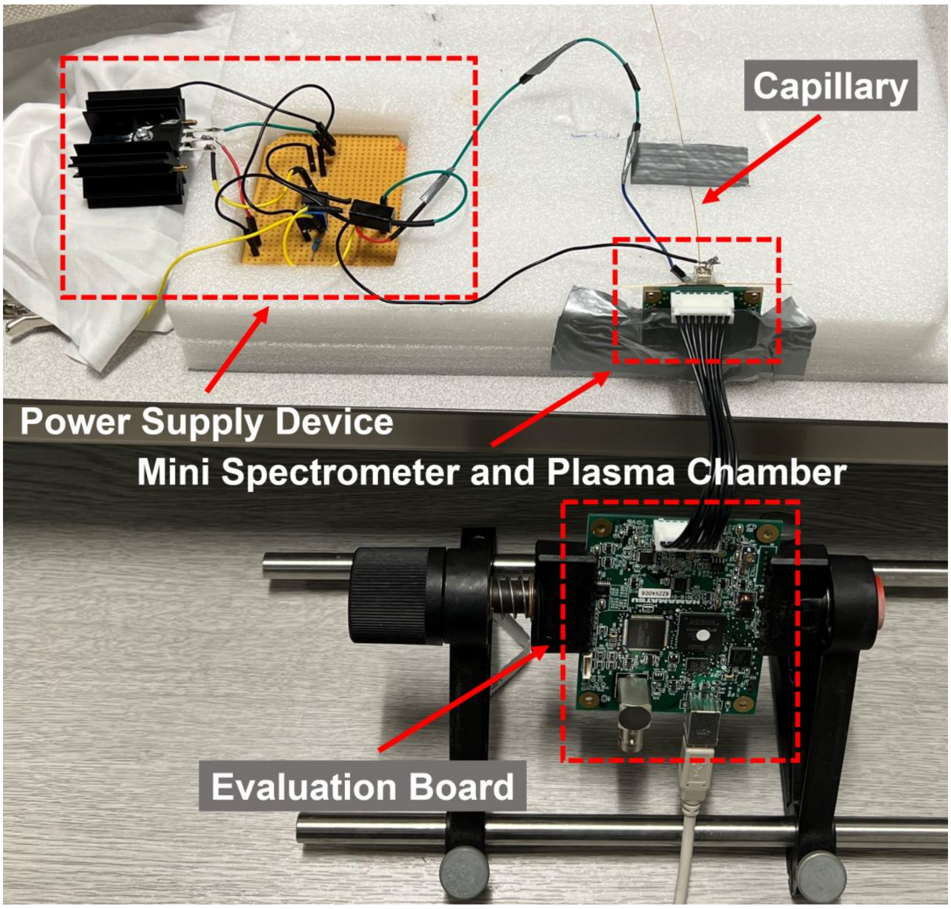

Figure S2. Image of the μHDBD-OES system. The power supply is the same as our previously reported work.[1] The operation of mini spectrometer requires it to be coupled with a Hamamatsu C13016 evaluation board.

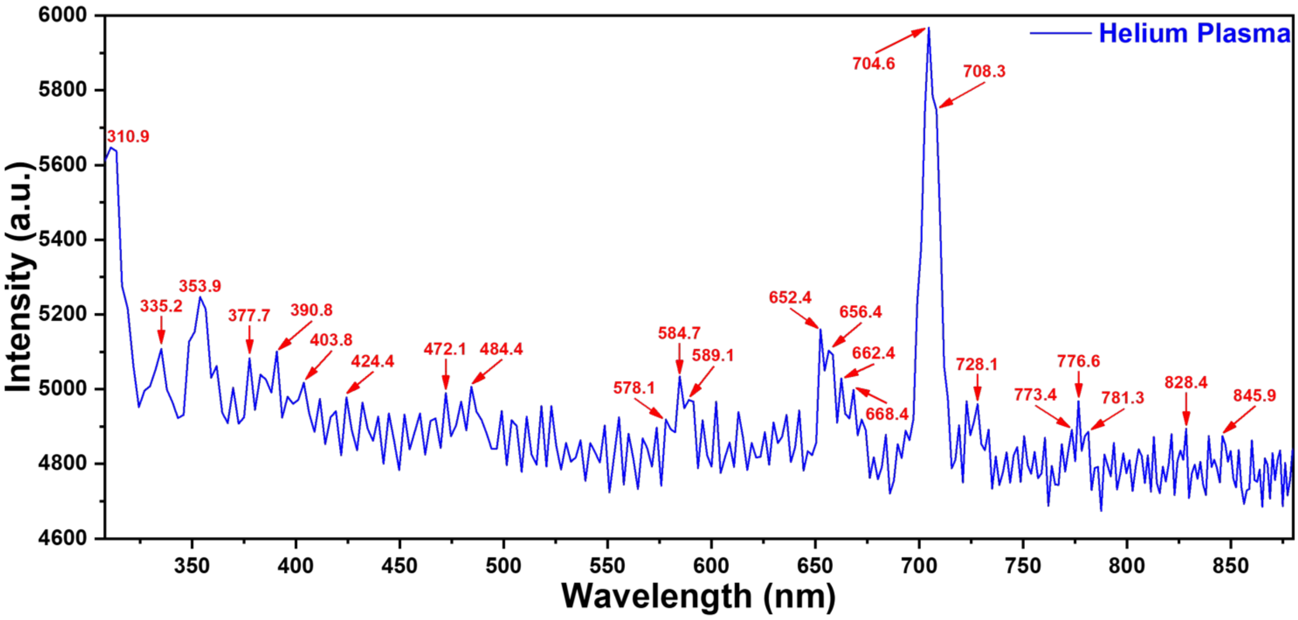

Figure S3. Optical emission spectrum of helium plasma in the range of 308 nm - 880 nm with the major peak values labelled on the graph (injection volume: 0.2 µL and split ratio: 200:1).

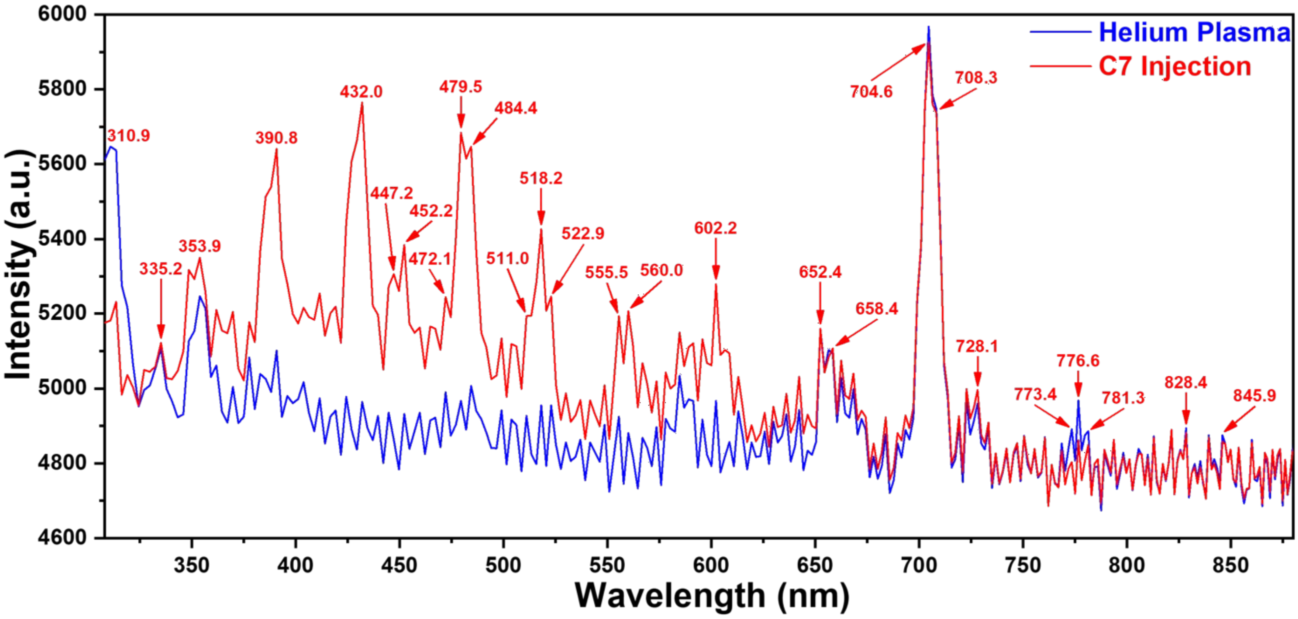

Figure S4. Optical emission spectrum of C7 in the range of 308 nm - 880 nm with the major peak values labelled on the graph (injection volume: 0.2 μL and split ratio: 200:1).

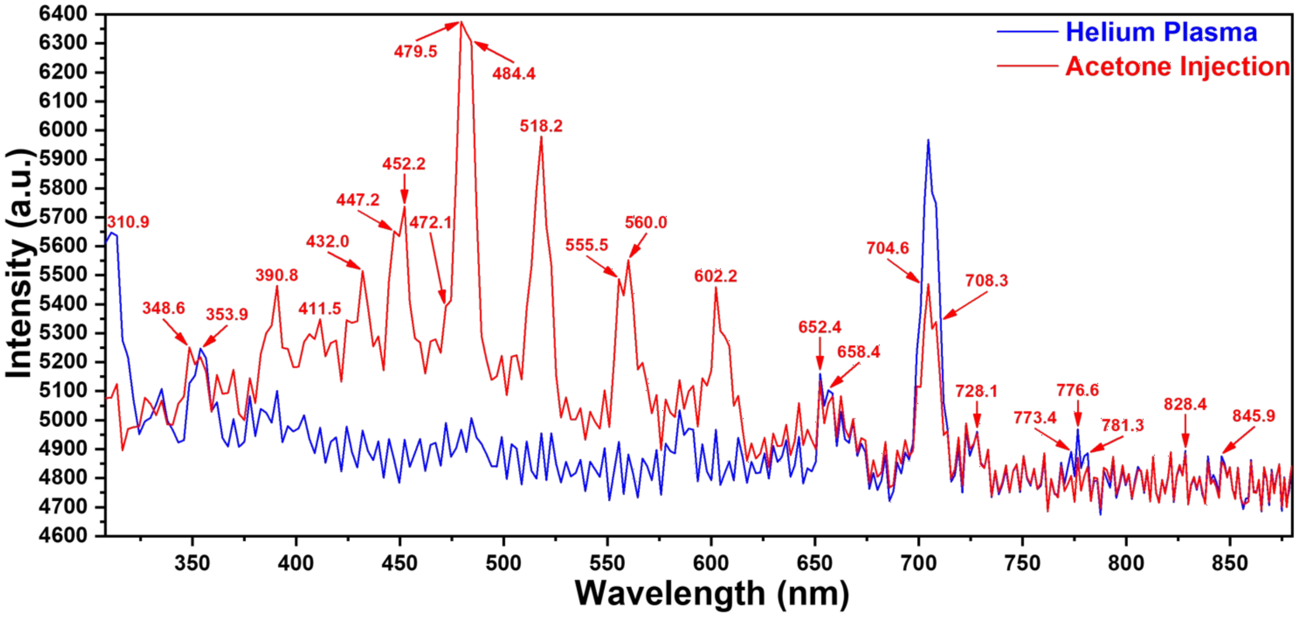

Figure S5. Optical emission spectrum of acetone in the range of 308 nm - 880 nm with the major peak values on the graph (injection volume: 0.2 µL and split ratio: 200:1).

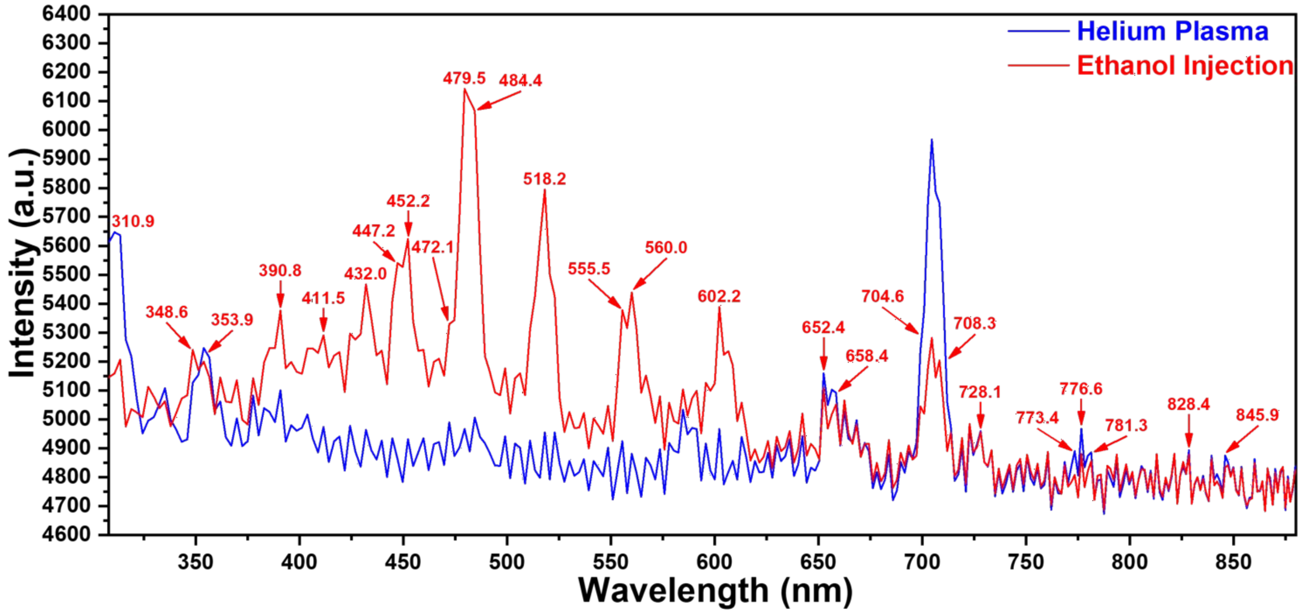

Figure S6. Optical emission spectrum of ethanol in the range of 308 nm - 880 nm with peak values labelled on the graph. (injection volume: 0.2 µL and split ratio: 200:1).

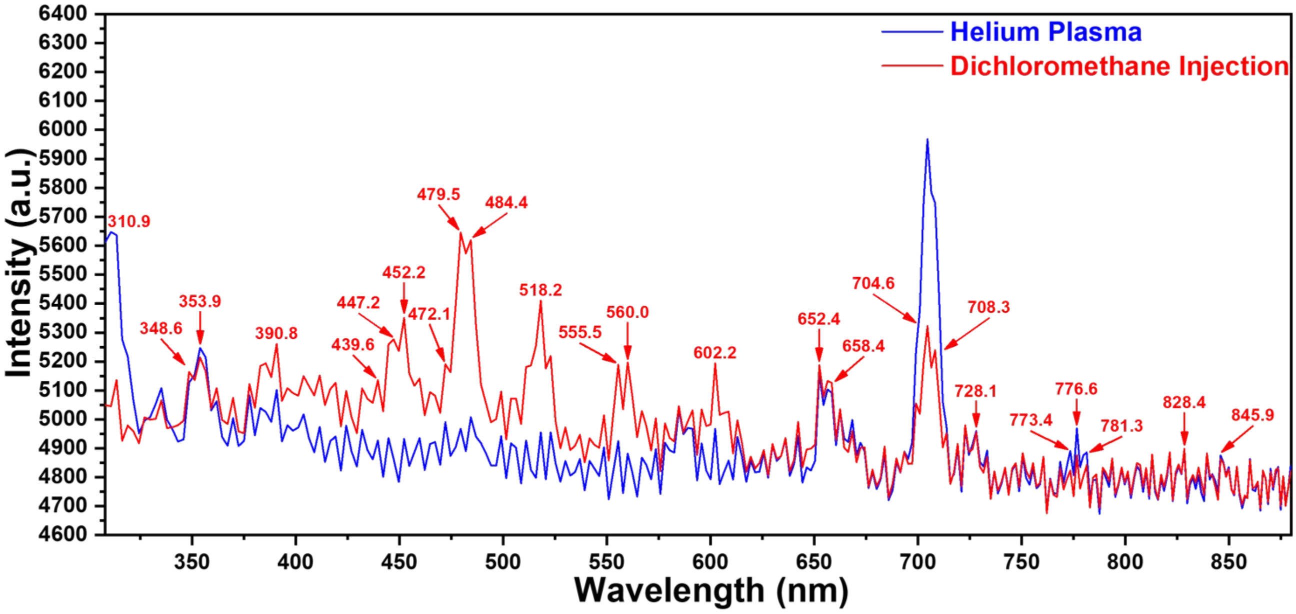

Figure S7. Optical emission spectrum of dichloromethane in the range of 308 nm - 880 nm with the major peak values labels on the graph. (injection volume: 0.2 µL and split ratio: 200:1).

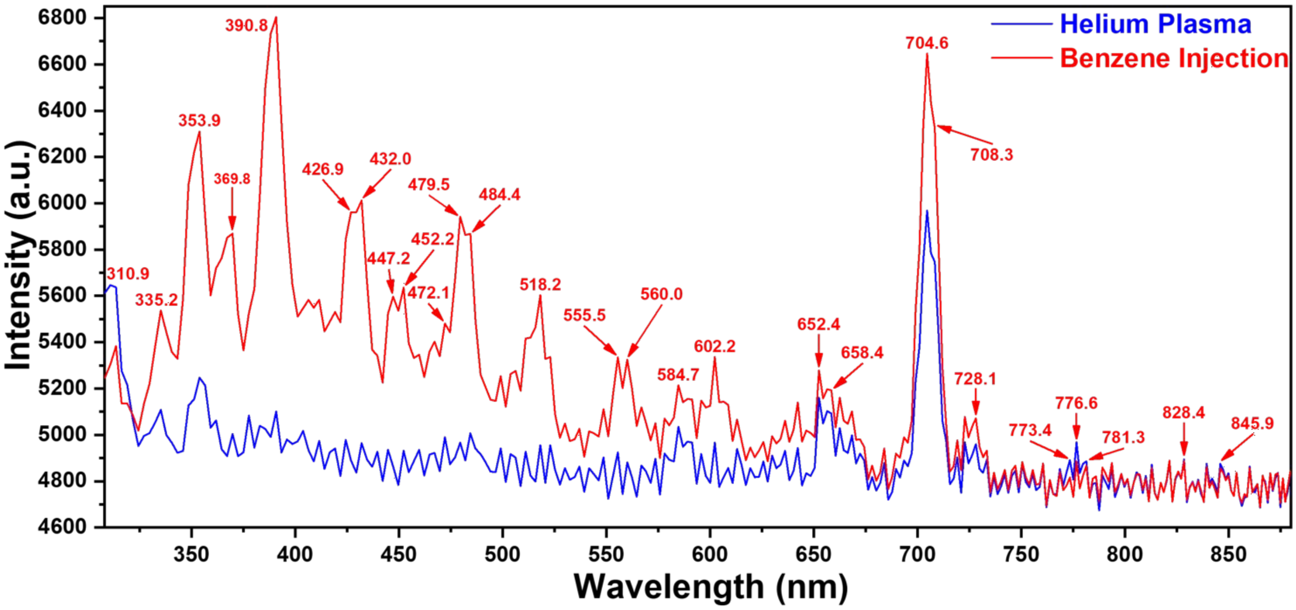

Figure S8. Optical emission spectrum of benzene in the range of 308 nm - 880 nm with peak values labelled on the graph. (injection volume: 0.2 µL and split ratio: 200:1).

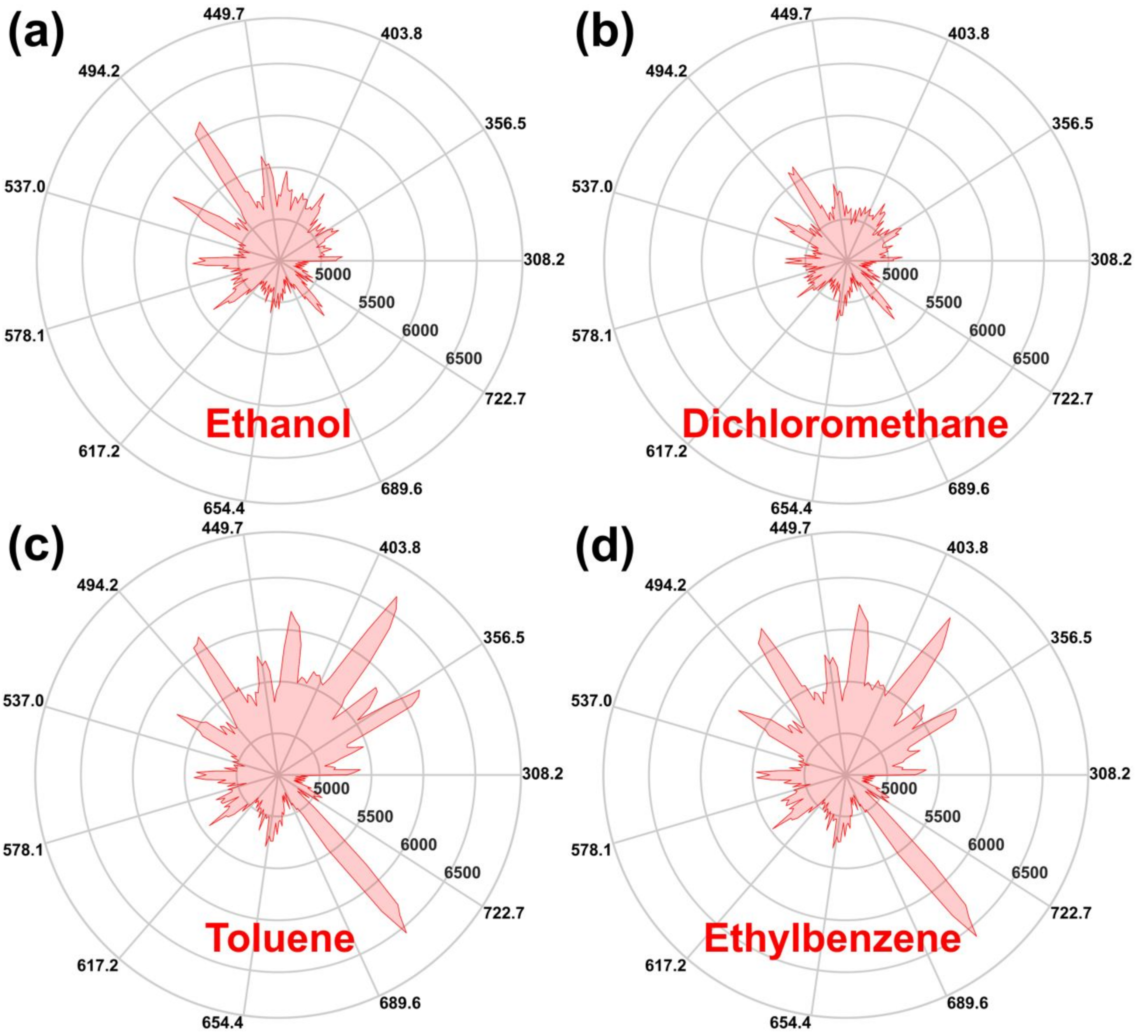

Figure S9. Emission spectra radar plots from 308.2 nm to 752.2 nm corresponding to Figures 3-5. (a) ethanol, (b) dichloromethane, (c) toluene, and (d) ethylbenzene. The ring axis represents wavelength (nm) and the polar axis is intensity (a.u.).

References

(1) Mao, J.; Liu, L.; Atwa, Y.; Hou, J.; Wu, Z.; Shakeel, H. Colorimetric Signal Readout for the Detection of Volatile Organic Compounds Using a Printable Glass-Based Dielectric Barrier Discharge-Type Helium Plasma Detector. *ACS Meas. Sci. Au* **2023**, *3* (4), 287−300.
